# Benchmarks for interpretation of QSAR models

**DOI:** 10.1186/s13321-021-00519-x

**Published:** 2021-05-26

**Authors:** Mariia Matveieva, Pavel Polishchuk

**Affiliations:** Institute of Molecular and Translational Medicine, Faculty of Medicine and Dentistry, Palacky University, University Hospital in Olomouc, Hnevotinska 5, 77900 Olomouc, Czech Republic

**Keywords:** QSAR model interpretation, Benchmark data set, Synthetic data set, Interpretability metrics, Atom contributions, Graph convolutional neural networks

## Abstract

Interpretation of QSAR models is useful to understand the complex nature of biological or physicochemical processes, guide structural optimization or perform knowledge-based validation of QSAR models. Highly predictive models are usually complex and their interpretation is non-trivial. This is particularly true for modern neural networks. Various approaches to interpretation of these models exist. However, it is difficult to evaluate and compare performance and applicability of these ever-emerging methods. Herein, we developed several benchmark data sets with end-points determined by pre-defined patterns. These data sets are purposed for evaluation of the ability of interpretation approaches to retrieve these patterns. They represent tasks with different complexity levels: from simple atom-based additive properties to pharmacophore hypothesis. We proposed several quantitative metrics of interpretation performance. Applicability of benchmarks and metrics was demonstrated on a set of conventional models and end-to-end graph convolutional neural networks, interpreted by the previously suggested universal ML-agnostic approach for structural interpretation. We anticipate these benchmarks to be useful in evaluation of new interpretation approaches and investigation of decision making of complex “black box” models.

**Supplementary Information:**

The online version contains supplementary material available at 10.1186/s13321-021-00519-x.

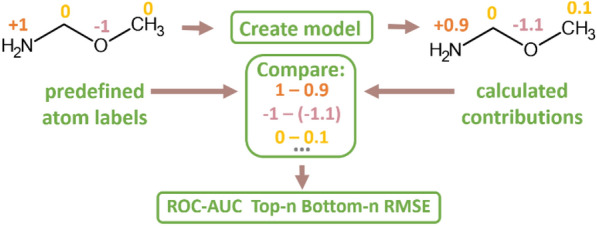

## Introduction

Interpretation of QSAR models is useful to understand the complex nature of biological or physicochemical processes. Information discovered by interpretation can be used to optimize structures of compounds, reveal structure–property trends or perform knowledge-based validation of models. Instance-based interpretation reveals contributions of atoms or fragments within individual molecules to identify most favorable or unfavorable motifs to consider subsequent modifications. Dataset-wide interpretation helps to establish contributions of fragments across different molecules of a data set to rank them and reveal general structure–property trends [1]. Interpretation of QSAR models became even more important in recent years after appearance of numerous deep learning approaches and their introduction to everyday practice [2]. It is important to understand decision making of such “black box” models to increase confidence in these models and retrieve useful knowledge [3].

Numerous approaches have been developed to interpret QSAR models. All approaches can be split in two categories: those which are applicable to particular machine learning models and those which are applicable to any models (machine learning method agnostic, ML-agnostic) (Table 1). Interpretation approaches can also be classified by the level of interpretation: feature-based or structural interpretation. In feature-based approaches contributions or importances of individual features/descriptors are calculated. This information can already be useful if descriptors are interpretable per se. Contributions of interpretable descriptors can be mapped back onto structure to reveal favorable/unfavorable substructures. Structural interpretation directly gives contributions of particular motifs skipping the step of calculation of descriptor contributions.

**Table 1:** QSAR interpretation approaches

	ML-dependent	ML-agnostic
Feature-based	Regression coefficients Rule extraction Layer-wise relevance propagation (LRP) CAM, GRAD-CAM	Sensitivity analysis Partial derivatives Feature importance by permutation Integrated gradients Shapley sampling values
Structural	Attention weights of attention-based (graph) neural networks	Universal approach of structural Interpretation Similarity maps Computational matched molecular pairs/series

Modern deep learning approaches frequently use end-to-end modeling and create own internal representation that makes many of commonly used approaches not applicable. This requires specific interpretation approaches developed for these methods [4]. There are multiple post hoc interpretation approaches developed specifically for neural network models, e.g., Layer-wise Relevance Propagation (LRP) [5], DeepLift [6], CAM [7], GRAD-CAM [8], etc. Some deep learning approaches are interpretable by design, e.g., attention-based neural networks [9]. Weights assigned by the attention layer can be interpreted as importances of corresponding features. In graph-based networks these features can be atoms and thus importance of individual atoms within a molecule can be established. ML-agnostic interpretation approaches can be applied to modern deep learning models too. Examples of feature-based approaches include Integrated Gradients [10], Shapley values [11, 12]. To the best of our knowledge, structural ML-agnostic interpretation methods haven’t been yet studied in application to deep neural networks (in QSAR field).

Despite the fact that multiple interpretation approaches have been developed and new ones constantly appear there are no suitable benchmarks to evaluate their applicability to interpretation of QSAR models. Often authors demonstrate applicability of their interpretation approaches on well-studied end-points like lipohilicity, solubility or toxicity where relevant patterns are well known [13]. Interpretation is usually performed for pre-defined motifs or on a limited number of considered examples [13–15]. For example, authors visually inspect a subset of molecules and compare calculated contributions with expert knowledge. Such non-systematic evaluation can be biased by a human expert and the choice of inspected molecules. Real data sets may have hidden biases which are difficult to control, some properties may depend on multiple factors or the response can be caused by different mechanisms of action. All these issues complicate proper validation of interpretation approaches based on real-world examples.

Synthetic data sets are more reasonable to evaluate interpretation approaches; they can be designed in such a way that end-point values are pre-defined according to some logic, e.g., presence or absence of chemical patterns combined by Boolean operators determining compounds’ activity (classification case). In regression case, the activity can be calculated as the sum of pre-defined atomic/fragment contributions. These data sets can be suitable to investigate the ability of models to capture the introduced logic and the ability of interpretation approaches to retrieve it. Two recent studies attempted using artificial data sets. The study by Sheridan was mainly focused on comparison of interpretation of models built using different conventional descriptors and machine learning methods [16]. The author used similarity maps for model interpretation which provided atom contributions (colors) [17]. Besides real-world datasets, two artificial, “idealized” ones were utilized. Both represented simple additive properties: heavy atom counts and the number of negative charges in compounds at pH 7.4. Regarding the impact of descriptors (D) and models (Q) on interpretation quality the author concluded, that “… one has to have a very high cross-validated predictivity to recover those expected colors, and not all D/Q combinations are suitable.”

Another study utilized Integrated Gradient interpretation method [10]. Authors used graph convolutional models and their interpretation produced atomic contributions. They created 16 synthetic classification data sets. Compounds were retrieved from ZINC database and satisfied particular positive or negative SMARTS patterns combined by Boolean operators. The goal of the study was investigating the ability of models to retrieve atoms corresponding to these positive and negative patterns. It was demonstrated that models could not always recognize true atoms correctly. Unfortunately, authors did not provide data sets to enable comparative studies. Besides, those data sets represented only one possible scenario of structure–property relationship where the property of compounds depended on local chemical context encoded by SMARTS. Regression tasks were not studied. In their most recent study the same authors demonstrated on similar synthetic data sets that not all interpretation methods, which were developed to interpret neural network, are suitable to retrieve structure–property relationships captured by these models. It was demonstrated that integrated gradients and class activation maps performed consistently well across multiple model types whereas GradInput, GradCAM, SmoothGrad and attention mechanism performed poorly [18]. This demonstrates that not all interpretation approaches are suitable to retrieve structure–property relationships from machine learning models and therefore proper validation of interpretation approaches is required.

The aim of this study was creation of synthetic data sets with pre-defined patterns determining end-point values and with control over possible biases. Development of these data sets will enable systematic evaluation of interpretation approaches to validate their ability to retrieve structure–property relationships captured by models, because calculated contributions of atoms or fragments can be compared with expected values determined by the incorporated logic (“ground truth”). We developed regression and classification data sets, which represent different logics and levels of complexity of end-points:

- Simple additive end-points, where specific contributions were assigned to individual atoms and the sum of atom contributions determined compound property.
- Additive end-points depending on a local chemical context, where contributions were assigned to groups of atoms and their sum determined the property value of a compound. This is related to molar refractivity or lipophilicity modeling, where group contribution methods are successfully applied [19, 20].
- Pharmacophore-like settings, where compounds were labeled as “active” if they had a specific 3D pattern. For simplicity we chose a two-point 3D pharmacophore. This case is the most similar to real problems, where property depends on distant features and their mutual orientation.

We also proposed metrics to quantitatively estimate performance of interpretation approaches. To demonstrate that the data sets and the metrics are suitable we applied previously developed *universal interpretation approach* [14], because it is ML-agnostic and allows to calculate the contribution of any atom or group of atoms. We applied it to multiple models built using conventional binary and count-based fingerprints and conventional machine learning methods and evaluated interpretability of these models. We also implemented this approach for graph convolutional neural networks (GC) as a part of Deepchem [21] and for the first time compared interpretability of GC with conventional QSAR models.

## Materials and methods

### Design of synthetic datasets

We created six data sets selecting compounds from the ChEMBL23 database (Table 2), which was used as a source of chemically relevant structures. Structures of all compounds were standardized, duplicates were removed, as were compounds with a MW > 500. For all retained compounds we assigned contributions to atoms or groups of atoms according to the rules described below, and then we calculated “activities” of compounds. To design regression data sets we assigned sampling probabilities to compounds so that their “activities” would resemble normal distribution. After that we randomly selected compounds from the pool with those probabilities. This resulted in distributions close to normal (Additional file 1: Figure S1). To design balanced classification data sets we randomly selected equal number of compounds belonging to each class.

**Table 2:** Synthetic data sets to benchmark interpretation of QSAR models

Dataset	Property type	End-point	Train/test set size	Expected atom contribution
N	Regression	Sum(N)	6995/2999	Nitrogen atoms: 1; others: 0
N − O	Regression	Sum(N) − sum(O)	6893/2969	Nitrogen atoms: 1; Oxygen atoms: − 1; others: 0
N + O	Regression	(Sum(N) + sum(O))/2,where sum(N) = sum(O)	7000/3000	Nitrogen and Oxygen atoms: 0.5; others: *0*
Amide_reg	Regression	Sum(NC=O)	7000/3001	Any atom of amide groups: 1; others: 0
Amide_class	Classification	Active: if sum(NC=O) > 0; inactive: if sum(NC=O) = 0	6998/3000	Any atom of amide groups: 1; others: 0
Pharmacophore	Classification	Active: at least one conformer with exactly one pharmacophore match (same two atoms in all conformers); inactive: no pharmacophore matches for all conformers; pharmacophore match: HBD and HBA 9–10 Å apart	7000/3000	Atoms which are HBA or HBD of the pharmacophore: 1; others: 0

Alternative is reasonable for interpretation methods that meet “summation to delta” property (see “Design of synthetic datasets”)

Three data sets represented simple additive properties. Patterns were defined as occurrence of certain atoms. The end-point of the first dataset (N data set) was the sum of nitrogen atoms. Thus, the expected contributions of nitrogen atoms were 1 and all other atoms—0. The end-point of the second dataset (N − O data set) was the sum of nitrogen atoms minus the sum of oxygen atoms. Thus, oxygen represented a negatively contributing pattern. Expected contribution of any nitrogen was 1, any oxygen − 1, and all others 0. The end-point of the third dataset (N + O dataset) was the sum of nitrogen and oxygen atoms divided by two. The number of nitrogen and oxygen atoms in a molecule was strictly equal. Thus, two positively contributing patterns were co-occurring and both contributed equally to the target property. This represents a specific case to verify how a model treats correlated patterns and how this affects interpretation output. Modeling algorithm can treat nitrogen and oxygen as equally important or select only one of them as important feature. Both these cases will result in correct predictions. If the model randomly prioritizes one of correlated features than rebuilding the model may result in different interpretation output. The same may happen if correlated features are removed before model building and during analysis of interpretation outcomes the discarded features are not considered. Depending on which scenario will be realized, the interpretation output may be incomplete and misleading.

We repeated the sampling procedure described above several times and calculated the correlation of selected atomic patterns with other elements to ensure that there is no explicit bias in data sets. Correlations varied a little between different runs and we chose data sets with lowest observed correlations (Additional file 1: Table S1). However, this does not guarantee that there are no correlations with more complex patterns.

Two other data sets represented additive end-points depending on local chemical context: they were collected independently and consisted of different compounds. The end-point of the first data set was the number of amide groups encoded with SMARTS NC=O. Thus, this was a regression task. The second data set was a classification one, where compounds were assigned active if they had at least one amide pattern and inactive otherwise. The expected contribution of any atom of an amide group for both data sets was set to 1, because upon removing of such an atom the whole pattern disappears. This disappearing should result in the decreasing of a predicted property value by 1 for the regression data set. In the case of the classification task, if a compound contains multiple amide groups, an issue may occur, because there is no single group which determines the activity. This may create complications for interpretation analysis and we will investigate this issue specifically.

The last data set was designed based on a pharmacophore hypothesis and represents property, depending on whole-molecule context. Compounds were labeled as active if at least one of their conformers had a pair of an H-bond donor and an H-bond acceptor 9–10 Å apart. If the pattern occurred in more than one conformer of a molecule, this had to be the same pair of atoms. Therefore, actives contained exactly one pharmacophore pair consistent across all conformers. If this pattern was absent in all conformers a compound was labeled inactive. Compounds with multiple pharmacophore pairs were excluded to avoid ambiguity in subsequent interpretation. We generated up to 25 conformers for each compound using RDKit. H-bond donors and acceptors were labeled using *pmapper* software [22]. We ensured that distributions of H-bond donors and acceptors in active and inactive classes were similar (Additional file 1: Figure S2). Atoms which were true pharmacophore centers had expected contribution 1; all other atoms—0. This example is closest to a real case scenario. However, we used a two-point pharmacophore to simplify modeling and interpretation. Using more complex pharmacophores with more features may require 3D descriptors to properly distinguish spatial arrangement of compounds and this could introduce additional difficulty to modeling using 2D representation. This can be implemented in future.

We verified that all data sets are representative of the source database and distributed similarly to it (Additional file 1: Figure S3). To create training and primary test sets all data sets were randomly split in a ratio 70/30. All datasets are provided in the repository https://github.com/ci-lab-cz/ibenchmark.

### Extended test sets

To reveal possible weaknesses in data sets and challenge the generalization ability of trained models we created an extended test set for each task. Structures from primary test sets were subject to small perturbations. This was done by applying the *mutate* operation implemented in the CReM tool [23]. We used the previously generated fragment database based on compounds from ChEMBL22 having maximum synthetic complexity score 2.5 [23, 24]. This database stores fragments and their local chemical context (atoms within a given radius from corresponding attachment points of a fragment). Fragments occurring in the same context are interchangeable and result in chemically valid structures. Filtering compounds used for fragmentation by their synthetic complexity result in more synthetically feasible and reasonable structures [24]. This has less importance in the present study but may prevent from appearing of unusual or “ugly” motifs in generated compounds. We chose neighborhood radius 3 and made all possible replacements of groups of up to three atoms with other groups of up to three atoms from the database. For each primary test set we generated around 300,000 new analogues and assigned end-point values to each compound using the same rules as for the corresponding data set (Table 2). The extended test sets provided more diverse examples of chemical space represented by primary test sets.

### Descriptors and model building

We employed the following descriptors: atom-pair fingerprints which enumerate pairs of particular atoms at the topological distance from 1 to 30 (AP), Morgan fingerprints which enumerate atom-centered substructures of radius 2 (MG2), RDK fingerprints which enumerate all possible substructures with atom count from 2 to 4 (RDK) and topological torsion fingerprints which enumerate all possible linear substructures with four atoms (TT). AP, MG2 and RDK fingerprints were also used in their binary (bit vector) form of length 2048. The corresponding binary fingerprints are denoted bAP, bMG2, and bRDK. All fingerprints were calculated using RDKit [25].

QSAR models were built using Random Forest (RF), Partial Least Squares (PLS), Gradient Boosting Machine (GBM) and Support Vector machine with Gaussian kernel (SVM) from *Scikit-learn* Python package [26]. Hyper parameters were optimized by the grid search in the course of five-fold cross-validation. We used SPCI software which automates overall modeling workflow and interpretation [27]. Graph convolutional neural network models (GC) were built using DeepChem [21]. This approach does not require external fingerprints and learn internal representation of molecules in the course of modeling. We used the default architecture with 2 graph convolutional layers, each of size 64 and *a GraphPool* layer after each convolution (*GraphPool* performs max pooling on each atom’s neighborhood). Output from the *GraphPool* layer was fed to “atom-level dense layer” (size 128) and global sum pooling *(GraphGather)* followed by linear or logistic regression layer depending on the task (regression, classification) (Additional file 1: Figure S4). We didn’t apply batch normalization. GC models can be considered as models trained on “learnable” Morgan fingerprints of radius 2. For training of GC models validation subsets (15%) were separated from training sets to tune model hyper parameters. We also trained 1-nearest neighbor (1-NN) models as baseline models to examine data set modelability [28]. Poor performance of 1-NN models would indicate that compounds are not easily distinguished within chosen descriptor space, indirectly indicating that data sets don’t have an obvious bias.

Predictive performance of models was assessed using the primary and extended test sets. Q^2^ and RMSE values were calculated for regression tasks and sensitivity, specificity and balanced accuracy were calculated for classifications tasks (Eq. 1–5).

1 $$Q^{2} = 1 - \frac{{\mathop \sum \nolimits_{i} \left( {y_{i,pred} - y_{i,obs} } \right)^{2} }}{{\mathop \sum \nolimits_{i} \left( {y_{i,pred} - \overline{y}_{obs} } \right)^{2} }}$$

2 $$RMSE = \sqrt { \frac{{\mathop \sum \nolimits_{i} \left( {y_{i,pred} - y_{i,obs} } \right)^{2} }}{N}}$$

3 $${\text{specificity}} = \frac{TN}{{TN + FP}}$$

4 $${\text{sensitivity}} = \frac{TP}{{TP + FN}}$$

5 $${\text{balanced accuracy}} = \frac{{{\text{sensitivity}} + {\text{specificity}}}}{2}$$

### Interpretation approach

We chose the *universal interpretation approach SPCI* because it allows to estimate a contribution of a single atom as well as an arbitrary group of atoms from any QSAR models [14]. We mainly focused our analysis on atom-based interpretation as the most popular and simple one, but in the case of the pharmacophore data set we also applied fragment-based interpretation. To estimate atom/fragment contribution an atom or a group of atoms is virtually removed from a compound and the end-point value is predicted for the remaining part of the compound. The contribution of the removed part is calculated as the difference between predicted value for the whole compound and the remaining part. To virtually remove atoms we used the scheme similar to that used by Sheridan in his study ﻿[16]. We replaced removed atoms with dummy atoms (with atomic number 0) and calculated descriptors. This resulted in appearance of new descriptors encoding dummy atoms, but these descriptors did not occur in the training sets, so the models ignored them during prediction. Thus, these atoms disappeared for models and prediction was made based on descriptors of the remaining part of the molecule. This scheme was implemented in *spci* software for RDKit-based fingerprints (https://github.com/DrrDom/spci) [27].

To estimate atom contributions from GC models we implemented the similar procedure to the described above. GC models convert each molecule into two input matrices at the preliminary featurization step: (1) atom basic features, and (2) connectivity information. To virtually remove an atom, we remove the corresponding row from the first matrix and adjust the connectivity table (the second matrix) respectively (Additional file 1: Figure S5). These modified matrices are supplied as input for a GC model which makes prediction. The contribution is calculated in the same way by subtracting the predicted end-point value for a compound with a virtually removed atom from the value predicted for the whole structure. We implemented this interpretation procedure in deepchem [29].

For regression models a calculated contribution is measured in the same units as studied end-points. For binary classification models the end-point used for calculation of atom/fragment contribution is probability of a compound to belong to the active class. Thus, the contribution represents the change in the probability of a molecule to belong to the active class upon removal of a particular atom/fragment. In this case the contribution has no units and can vary from − 1 to 1. An important note is that we used only training set for estimation of interpretation performance. This should give more accurate results than test set because prediction error for a training set is smaller.

### Interpretation quality metrics

The important ability of machine learning models is finding of relevant patterns which discriminate actives from inactives. Since we focused mainly on contributions of atoms we performed only instance-based interpretation. We considered each molecule individually and analyze the ability of interpretation methods to rank atoms in proper order, i.e., atoms with greater expected contributions should be ranked higher.

For atom-based interpretation we propose to calculate ROC AUC, top-n and bottom-n scores, and RMSE. Metrics (where applicable) are computed individually for positively contributing atoms (hereafter positive atoms) and for negatively contributing atoms (hereafter negative atoms). Positive atoms are atoms which increase activity (regression) or favor positive class prediction (classification). Negative atoms—vice versa.

ROC AUC is an integral metric which demonstrates how well relevant patterns (atoms) are ranked over others within a particular molecule. To get the final score we averaged AUC values for all considered molecules. In QSAR interpretation context this metric was first used by McCloskey et al. [10]. To calculate AUC for positive patterns (AUC^+^) we set all negative atoms’ labels to 0. Thus, AUC^+^ characterized how high positive patterns were ranked. To calculate AUC for negative patterns (AUC^−^) we set negative atoms’ labels to 1 and all others to 0. It worth noting that AUC cannot be calculated for molecules having no patterns pre-defined for a given dataset (expected contributions of all atoms are 0). Therefore, these molecules were not considered for the calculation of average AUC for individual data sets.

The weakness of ROC-AUC is that it is an integral measure and accounts for both relevant and irrelevant patterns. In practice it is more reasonable to find relevant features. To address this, we propose top-n score which is calculated as follows and should be more stringent:

$${\text{top{-}n score}} = \frac{{\mathop \sum \nolimits_{i} m_{i} }}{{\mathop \sum \nolimits_{i} n_{i} }},$$

where *n*_*i*_ is the total number of positive atoms in the i-th molecule, *m*_*i*_ is the number of positive atoms in *n*_*i*_ top ranked atoms according to their calculated contributions. For instance, if a molecule has two true patterns with expected contributions + 1 and interpretation retrieved only one of them among top two contributing patterns, the molecule will contribute n = 2 and m = 1 to the equation above. Top-n is an integral characteristic of a data set and varies from 0 to 1 (perfect interpretation). Analogously we calculated bottom-n score to estimate the ability to retrieve negative patterns.

Additionally, we calculated root mean square error (RMSE) of predicted contributions for each molecule and averaged them across molecules in a data set to estimate deviation of calculated contributions from the expected values. This is less important metric, because proper ranking is more practically valuable than exact estimation of contributions which are generally unknown in real cases. But RMSE should be helpful for benchmarking purposes because it allows to investigate decision making of models and interpretation methods from another point of view and may reveal other weaknesses or advantages not captured by other metrics.

The described metrics were implemented in the open-source repository to facilitate calculation of interpretation performance—https://github.com/ci-lab-cz/ibenchmark.

## Results

### Model performance

**Fig. 1:**
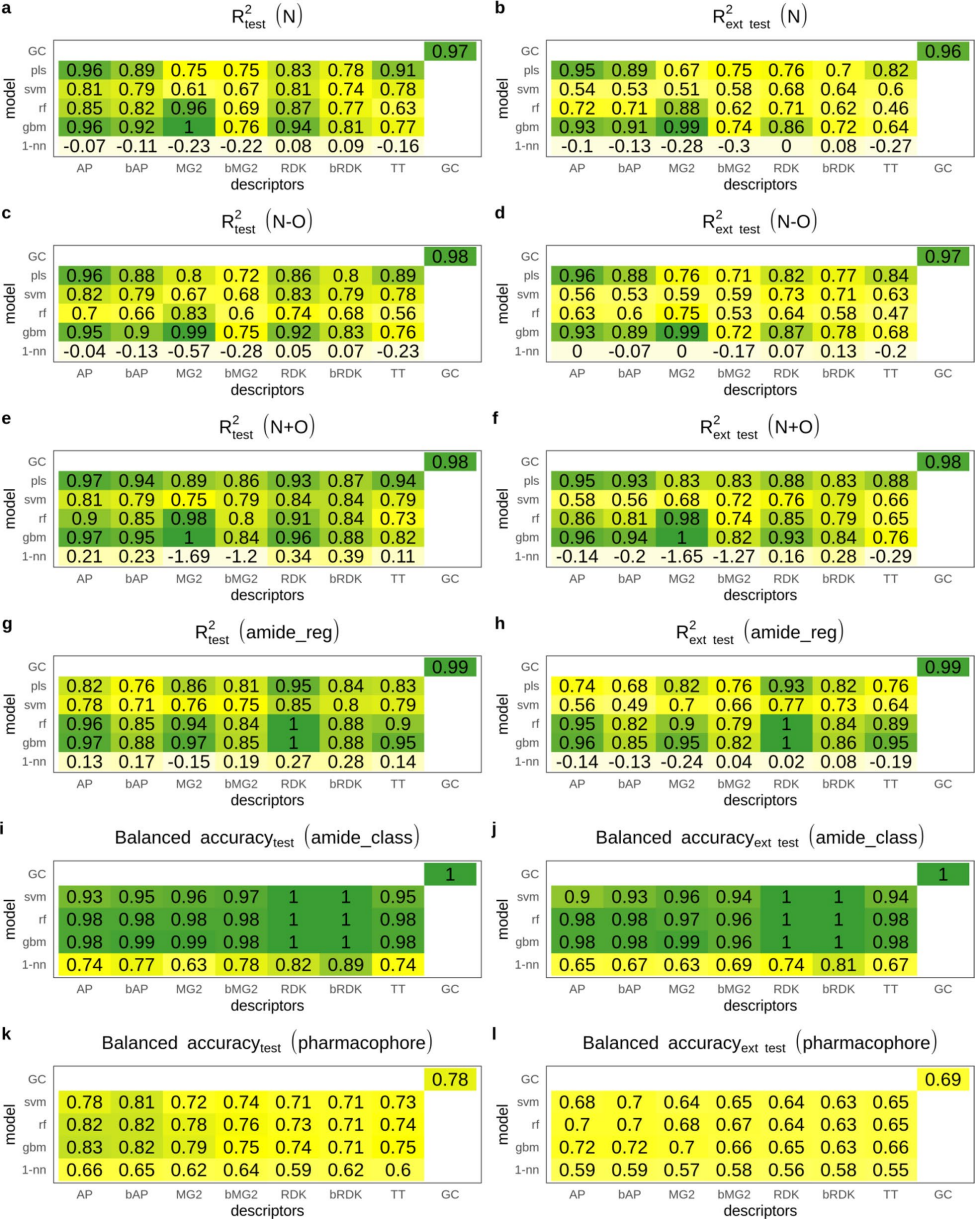
Performance of models on primary and extended test sets for regression and classification data sets

For all regression datasets baseline 1-NN models demonstrated poor results irrespective of descriptors and machine learning method used (Fig. 1). In almost all cases R^2^ was less than 0.3 for both test sets. In all cases performance of 1-NN models was significantly poorer than performance of other models. This indicates that these data sets do not have easily distinguishable patterns in chosen descriptor space and are not biased in that way.

Performance of models under study on regression data sets varied for both primary and extended test sets and depended on the combination of descriptors and machine learning method. However, there were at least several models with almost perfect predictions for each data set. GBM models trained on count-based Morgan fingerprints achieved consistently high performance on all regression tasks (R^2^ = 0.95–1.0, Fig. 1a–h). Models trained on binary Morgan fingerprints followed by binary RDK fingerprints achieved the lowest performance across all data sets irrespective of the machine learning method used. Expectedly, binary fingerprints resulted in less predictive models than corresponding count-based fingerprints. SVM demonstrated lower accuracy on all regression data sets that could be explained by RBF kernel chosen whereas studied activities were additive and could be captured by simpler linear models. Performance of models on extended test sets was lower than on primary tests, but for highly predictive models this difference was absent or minimal. These models recognized correct patterns and challenging them by structural perturbations did not compromise their predictive performance. Therefore, we concluded that regression data sets do not have a hidden bias. Lower performance of weak models on extended test sets suggests rather model fault than a data set bias.

Moderate performance of 1-NN models achieved for classification amide data set was expected, because an amide group is essentially distinguishable by fingerprints used. Performance of GBM, RF, SVM and GC models was much higher. Balanced accuracy was greater than 0.9 for both primary and extended test sets (Fig. 1i, j) suggesting that in all cases models were able to capture relevant patterns. The second classification task, the pharmacophore data set, was much harder because the models trained on 2D descriptors should capture the 3D pattern. The best baseline 1-NN model trained on AP descriptors had balanced accuracy 0.66. Models under study demonstrated moderate performance, but higher than that of corresponding 1-NN models (Fig. 1k, l). GBM, RF and SVM models trained on count-based and binary AP descriptors had the highest balanced accuracy (> 0.8) on the primary test set. There was a slight difference between performance on primary and extended test sets. Models which had higher performance on the primary test set had higher performance on the extended test set similarly to regression models.

GC models were among the best ones across all data sets confirming this modeling approach to be competitive to conventional ones in terms of predictive ability [30].

### Interpretation performance

#### N data set

**Fig. 2:**
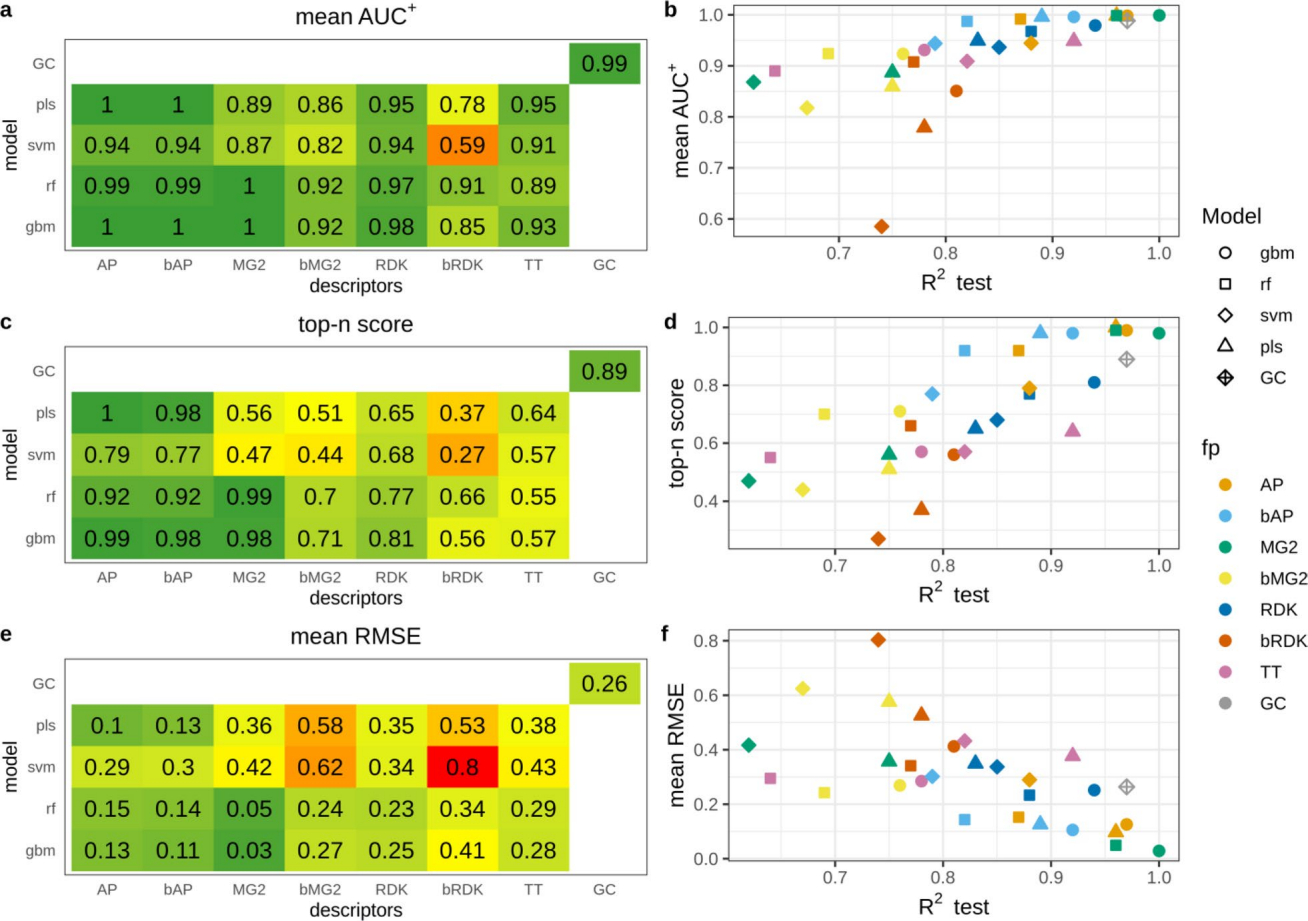
Interpretation performance of models trained on the N data set

High average AUC^+^ values observed in the majority of cases indicate that atoms were ranked correctly (Fig. 2a). For models having test set R^2^ > 0.81 average AUC^+^ was greater than 0.9 (Fig. 2b). There is a clear correspondence between model predictivity and the ability to rank atoms. However, there were few outliers, namely SVM and PLS models trained on binary RDK fingerprints. Their average AUC^+^ values were substantially lower than AUC^+^ of other models of comparable predictivity. AP, bAP and RDK fingerprints resulted in the highest average AUC^+^ irrespective of machine learning method. Count-based Morgan fingerprints with RF and GBM models had perfect average AUC^+^. The GC model also resulted in high accuracy in atom ranking (average AUC^+^ = 0.99).

Top-n score characterizes the ability to rank true atoms on top. This metric was more sensitive to the changes of R^2^ than AUC^+^ (Fig. 2c, d). The most predictive models had high top-n scores. These were PLS, RF and GBM models trained on AP and bAP fingerprints and RF and GBM models trained on count-based Morgan fingerprints (top-n = 0.92–1.0). GC model had somewhat lower score (0.89) followed by models trained on count-based RDK fingerprints (top-n = 0.65–0.81). Interestingly, GC model demonstrated top-n score lower than models having even lower predictive ability. For example, RF trained on bAP had R^2^ 0.82 and 0.71 for primary and extended test sets and top-n 0.92, whereas the GC model had R^2^ 0.97 and 0.96 and top-n 0.89. RMSE of calculated contributions is an absolute measure of interpretation accuracy. RMSE values varied in a wide range but were in good agreement with top-n. Therefore, we did not analyze them in detail. It is worth noting, that models trained on binary atom pairs had similar interpretation performance to those trained on count-based atom pairs, whereas the former had poorer predictive ability. For other pairs of corresponding binary and count-based fingerprints the latter always outperformed the former.

#### N − O data set

**Fig. 3:**
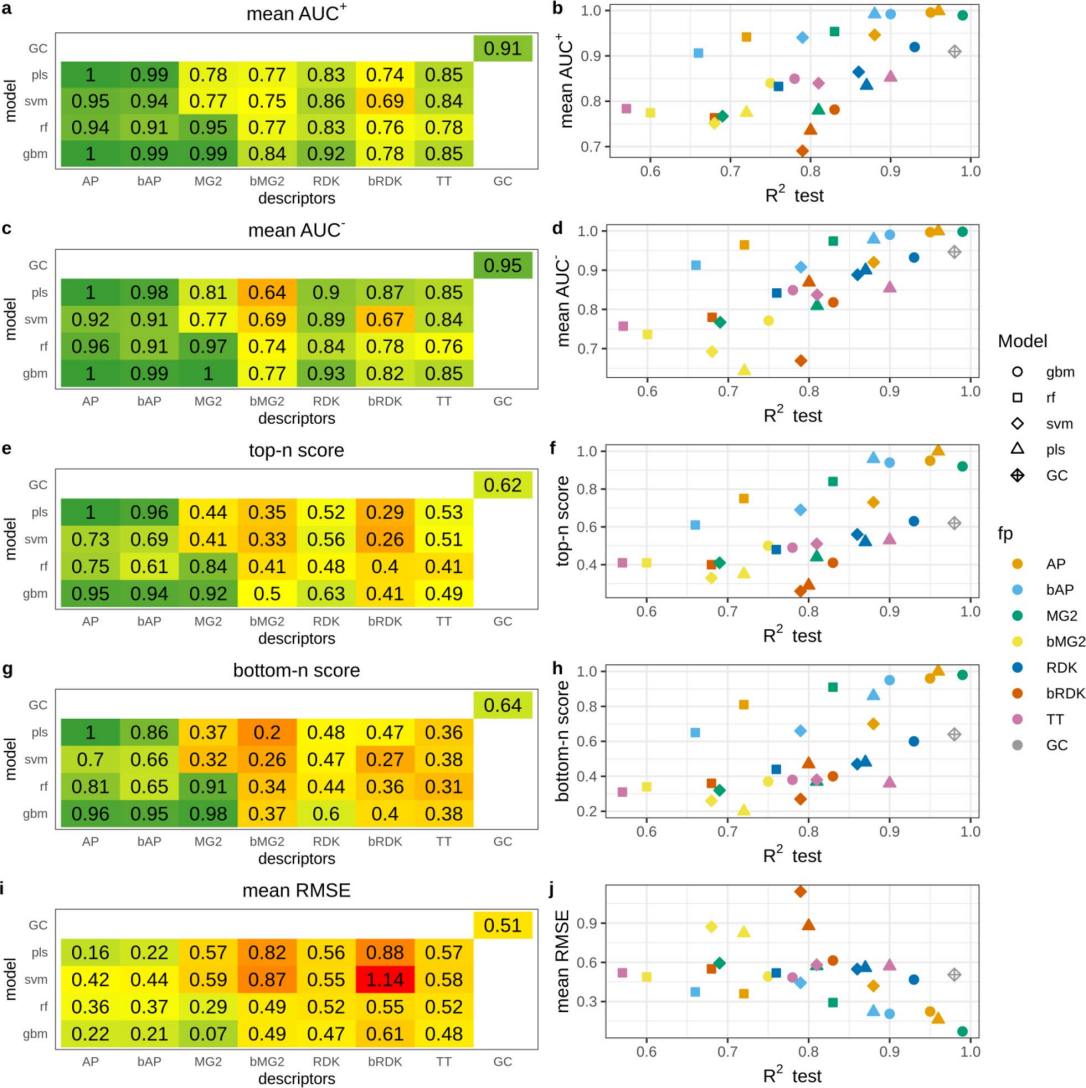
Interpretation performance of models trained on the N − O data set

This data set contained positive (nitrogen) and negative (oxygen) atoms. Therefore, we evaluated interpretation accuracy separately for each pattern. We found that overall ranking abilities for positive and negative patterns measured by AUC^+^/AUC^−^ were similar (Fig. 3a, c). The same agreement was observed for top-n and bottom-n scores (Fig. 3e, g). Thus, models were able to detect positive and negative patterns with comparable accuracy. The only outlier was PLS models trained on binary Morgan (AUC^+^  = 0.77, AUC^−^ = 0.64, top-n = 0.35, bottom-n = 0.2) and RDK fingerprints (AUC^+^  = 0.74, AUC^−^ = 0.87, top-n = 0.29, bottom-n = 0.47), showing relatively large discrepancy between positive and negative pattern recognition. Because of high correspondence in positive and negative pattern detection we will discuss only positive patterns.

The relationship between model predictive ability and interpretation accuracy was less stringent relatively to the N data set, but highly predictive models still resulted in high interpretation performance. However, models trained on AP and bAP fingerprints always resulted in high interpretation performance (AUC^+^  = 0.91–1.0, top-n = 0.61–1.0) regardless of their predictive ability (R^2^_test_ = 0.66–0.96). RMSE was in a good agreement with other metrics and we will not discuss it.

**Fig. 4:**
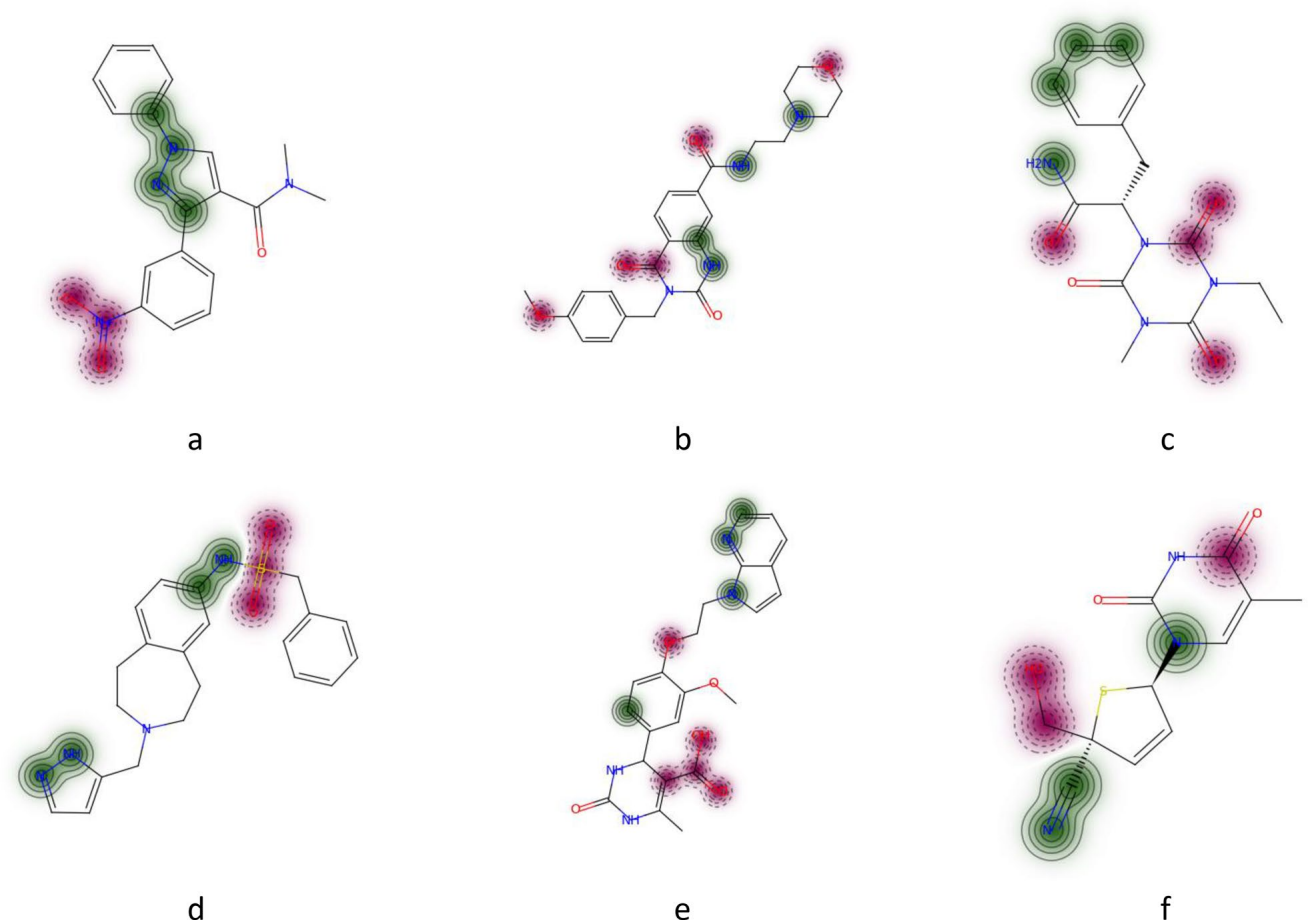
Top-scored (green) and bottom-scored (pink) atoms by the GC model for the N − O data set. The number of top and bottom highlighted atoms is equal to the total number of positive (nitrogen) and negative (oxygen) atoms in corresponding molecules

Interpretation of the GC model resulted in lower interpretation accuracy than that of other models of comparable or even lower prediction accuracy. The most striking difference was observed for top-n score which was 0.62 for the GC model, whereas corresponding values for PLS and GBM models trained on AP descriptors were 1.0 and 0.95. However, all these models had comparable predictive performance: R^2^ for the primary test set was 0.96, 0.96 and 0.97 for PLS, GBM and GC models, respectively (Fig. 3c, d). We inspected the interpretation results of the GC model for a randomly chosen subset of 100 molecules. There were several kinds of misinterpreted patterns (Fig. 4). Atoms neighboring to true ones often received greater contributions. For instance, carbon atoms in carbonyl and carboxyl groups or sulfur atoms in sulfonyl groups were often ranked on bottom (pink). Atoms attached to nitrogen were ranked on top (green) (Fig. 4a–f). Sometimes nitrogens in nitro groups were misinterpreted as negative (Fig. 4a). Aromatic carbons were frequently recognized as positive, though they were far from any nitrogen (Fig. 4c, e).

#### Regression amide data set

**Fig. 5:**
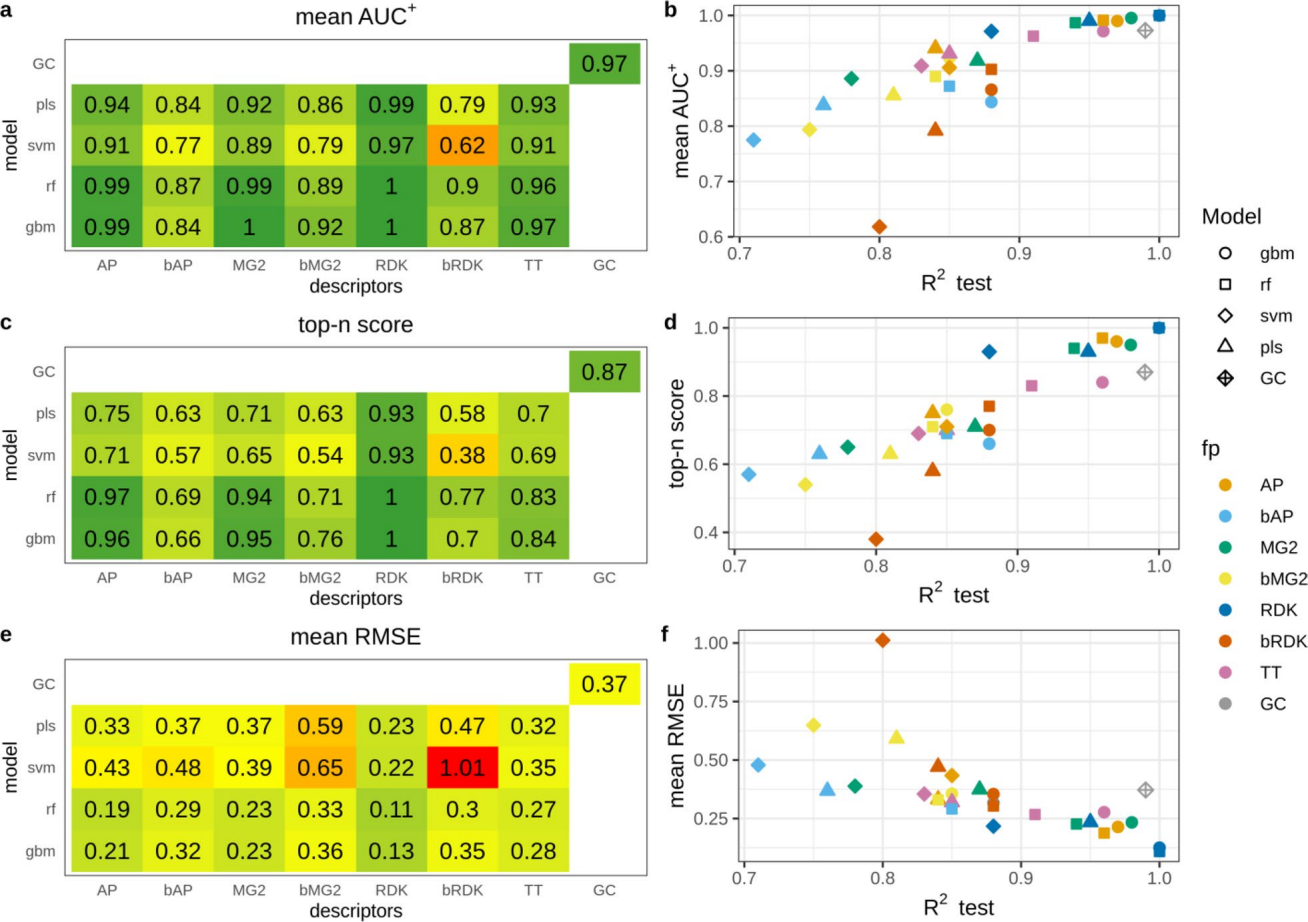
Interpretation performance of regression models trained on amide data set

The overall ranking ability of highly predictive models was very high and it decreased with decreasing of model R^2^_test_, but AUC^+^ remained above 0.8 (Fig. 5a, b). The overall trend was very similar to the N data set (Fig. 2b). SVM model trained on binary RDK fingerprints had very low interpretation accuracy (AUC^+^ = 0.62) close to random ranking (0.5) and this was the main outlier from the trend. The relationship between top-n score and model predictive ability was quite stringent with two noticeable outliers: SVM models trained on binary and count-based RDK fingerprints (Fig. 5c, d). While the former had relatively low score (0.38) the latter had the higher score (0.93) than other models with comparable predictive ability. It should be noted that models built on count-based RDK fingerprints were among the strongest in terms of interpretation accuracy, whereas predictive ability for some of them was relatively low. For example, SVM model trained on count-based RDK fingerprints had R^2^_test_ = 0.85, AUC^+^ = 0.97 and top-n = 0.93, while RF model trained on count-based Morgan fingerprints had much higher predictive ability (R^2^_test_ = 0.97), but comparable interpretation accuracy (AUC^+^ = 0.99, top-n = 0.94). It should be noted that in all cases count-based fingerprints outperformed binary, most probably due to the loss of information discriminating molecules with different number of true patterns. The GC model had slightly lower interpretation performance than models of comparable predictive ability, similarly to the case of the N − O data set.

#### Classification amide data set

**Fig. 6:**
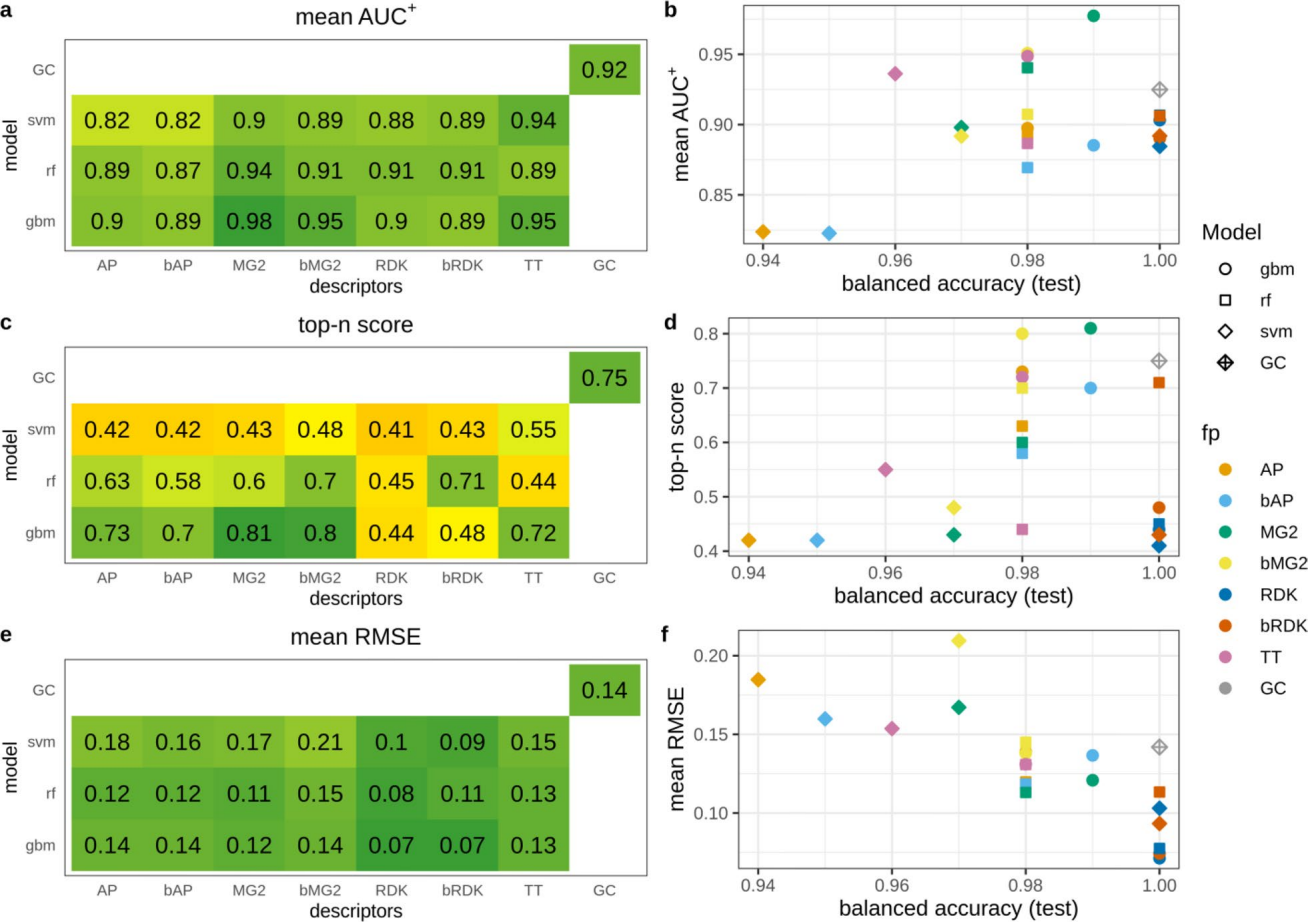
Interpretation performance of models trained on the classification amide data set

This data set was simple for modeling and all models achieved high balanced accuracy (≥ 0.93). However, overall ranking ability for atoms of amide groups varied in a wide range (AUC^+^ = 0.82–0.98) (Fig. 6a, b). The trend that models with better predictive ability demonstrate better interpretation accuracy was not observed. Models with R^2^_test_ = 1 (all models trained on both types of RDK fingerprints and the GC model) demonstrated relatively low ranking ability (AUC^+^ = 0.88–0.92). This was even more pronounced in the case of top-n score which was below 0.5 for most of these models (Fig. 6c, d), meaning that only half of true atoms were ranked on top. This is a consequence of the interpretation approach which virtually removed an atom and calculated the contribution of the removed part as the difference between predicted active class probabilities. If the property depends on the presence or absence of a particular pattern, but there are multiple such patterns in a molecule, then removing one of them will keep the remaining structure highly probable to be predicted active and the calculated difference will be small. We confirmed this by investigating interpretation accuracy for subsets of molecules having different number of true patterns using three models (Table 3). GBM model trained on MG2 descriptors had the highest interpretation performance. Average AUC^+^ values slowly decreased with increasing of the number of true patterns in molecules. Top-n score was more sensitive and substantially decreased for molecules having two amide patterns (from 0.98 to 0.69). In the case of GBM model trained on AP descriptors all interpretation metrics were more sensitive to the number of true patterns. For example, for molecules having two amide patterns AUC^+^ dropped from 0.96 to 0.77, and top-n score dropped from 0.89 to 0.58. Similar picture was observed for the GC model (Table 3).

**Table 3:** Interpretation performance of selected models calculated for subsets of molecules having different number of amide groups

Count of amide groups	GBM/MG2			GBM/AP			GC		
	Mean AUC^+^	Top-n	Mean RMSE	Mean AUC^+^	Top-n	Mean RMSE	Mean AUC^+^	Top-n	Mean RMSE
All	0.98	0.81	0.12	0.90	0.73	0.14	0.92	0.75	0.14
0	–	–	0.03	–	–	0.02	–	–	0.02
1	1	0.98	0.12	0.96	0.89	0.17	1	0.98	0.2
2	0.94	0.69	0.4	0.77	0.58	0.42	0.81	0.56	0.36
3	0.9	0.65	0.51	0.75	0.6	0.53	0.66	0.44	0.52
4	0.87	0.62	0.57	0.6	0.45	0.57	0.53	0.39	0.57
5	0.8	0.44	0.58	0.57	0.47	0.58	0.54	0.33	0.57
6	0.66	0.55	0.67	0.49	0.39	0.67	0.61	0.48	0.67

#### Pharmacophore data set

**Fig. 7:**
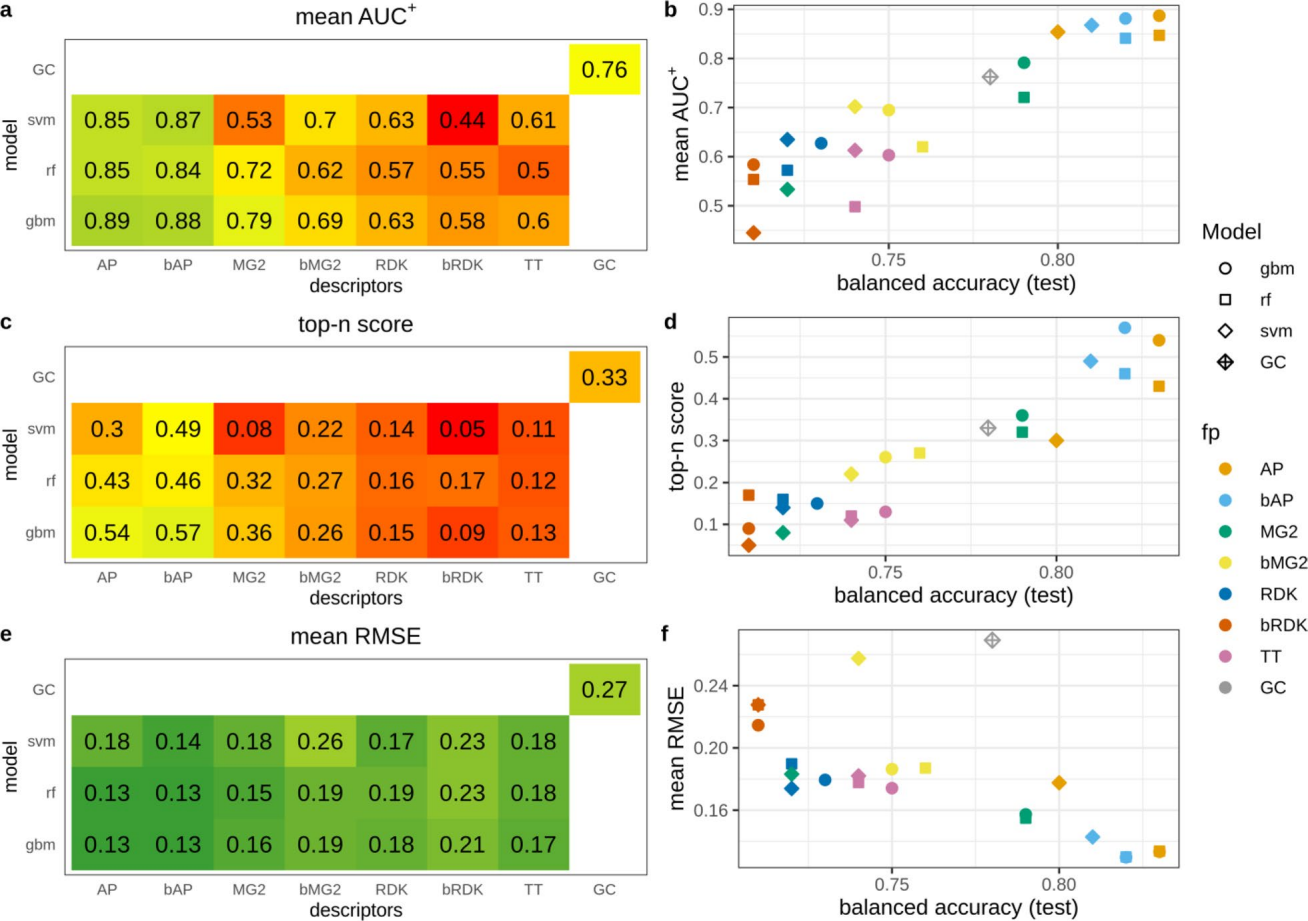
Interpretation performance of models trained on pharmacophore data set

The pharmacophore dataset was the hardest task and models achieved moderate balanced accuracy. Thus, it was expected that interpretation accuracy would be relatively low (Fig. 7). Interestingly, for this data set the correlation between model predictivity and interpretation accuracy was the most pronounced and predictive ability of conventional models mostly depended on descriptors type. Both types of atom pairs fingerprints resulted in the most accurate models followed by count-based Morgan fingerprints. All models built on atom pairs fingerprints demonstrated reasonably high ability to rank atoms (AUC^+^ = 0.84–0.89). This observation can be explained by the nature of the end-point–two specific atoms at a distance of 10–11A. Atom pairs were the only descriptors which could capture long distance interaction of features, because they took into account atoms up to 30 bonds apart. RF and GBM models trained on count-based Morgan fingerprints and the GC model had moderate overall raking ability (AUC^+^ = 0.7 = 0.79). Multiple models had average AUC^+^ values close to 0.5 or even lower, however predictive ability of all these models was moderate (R^2^_test_ ≥ 0.71). This suggests that models considered as acceptable according to their predictive ability may result in ranking ability of patterns close to random choice.

AUC metric may be particularly not suitable in this case because each molecule of the active class has only two true pharmacophore centers being ranked against all remaining atoms. This could result in artificially high AUC values if both true patterns were ranked close to the top but not on top. We expect top-n to be a more reasonable and stringent metric in this case. Models with the highest average AUC^+^ values had relatively low top-n scores 0.30–0.57 (Fig. 7c). This means that on average they identified only 30–57% of true pharmacophore centers within top 2 scored atoms. Most of other models had even lower performance. Because each active molecule had only two true pharmacophore centers, the top-n score was equivalent to the average percentage of true centers in top 2 atoms. To augment this value we calculated the average percentage of true centers in top 3 and top 5 atoms (Table 4). This is a common metric frequently used to measure accuracy of prediction of true active centers, for example sites of metabolism [31]. The results demonstrated that probability to find true pharmacophore center substantially increased with considering more atoms. For the best model GBM/AP the probability to find true pharmacophore centers in top 5 atoms reached 77%. For the GC model the increase was less pronounced, just to 50% (Table 4).

**Table 4:** Average percentage of indentified true pharmacophore centers in top scored atoms for the pharmacophore data set

Model	Top-2 (%)	Top-3 (%)	Top-5 (%)
GBM/AP	54	63	77
RF/AP	43	54	67
SVM/AP	30	41	63
GC	33	39	50

**Fig. 8:**
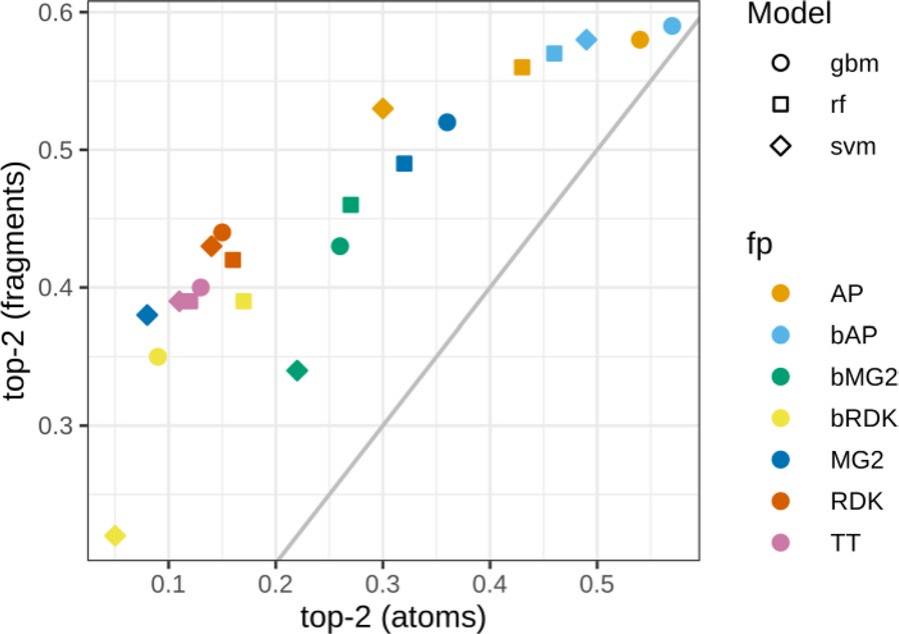
Top-2 score for atom- and frgament-based interpretation of models trained on the pharamcophore data set

Since atom-based interpretation resulted in relatively low performance we examined fragment-based interpretation performance. The motivation was to check whether selection of larger fragments (more than one atom) would help to better locate true centers at least approximately. We exhaustively fragmented training set molecules by breaking up to three bonds matching SMARTS [!#1]!@! = !#[!#1] using RDKit [25]. From resulting fragments, we kept only those of the size up to seven heavy atoms and the fragment size was limited to 40% of the total number of heavy atoms in a molecule. Effectively, since we did not break rings, this allowed us to estimate contributions of six-membered rings with one attached atom. To evaluate performance of fragment-based interpretation we chose top-2 metric calculated similarly to top-n metric for atoms. Top 2 scored fragments were chosen for each molecule and if both true centers were captured by these fragments the score was 1, if only one − 0.5, if none − 0. The scores were averaged among all molecules to get the final value. The metric top-2 for fragments was equivalent to top-n for atoms because each compound had exactly two true centers. Therefore, we plotted them together (Fig. 8). For models which demonstrated high performance in atom-based interpretation, performance of fragment-based interpretation was not substantially better. But models with relatively poor performance at the atom level demonstrated substantially better accuracy of identification of fragments comprising true centers. For example, GBM model trained on RDK descriptors could identify only 15% of true centers within top 2 scored atoms and 44% true centers within top 2 scored fragments.

RMSE has a little sense for classification models but we observed a clear relationship that RMSE values gradually increased with decrease of model predictive ability (Fig. 7f). The only outlier was the GC model. This could be explained by the fact that sigmoid activation function used on the output layer resulted in predicted probabilities substantially shifted towards 0 or 1 (Additional file 1: Figure S6). Thus, a small perturbation of a structure may result in substantial changes in the predicted probability for a molecule and, as a consequence, large calculated contribution.

#### *N* + *O dataset*

**Fig. 9:**
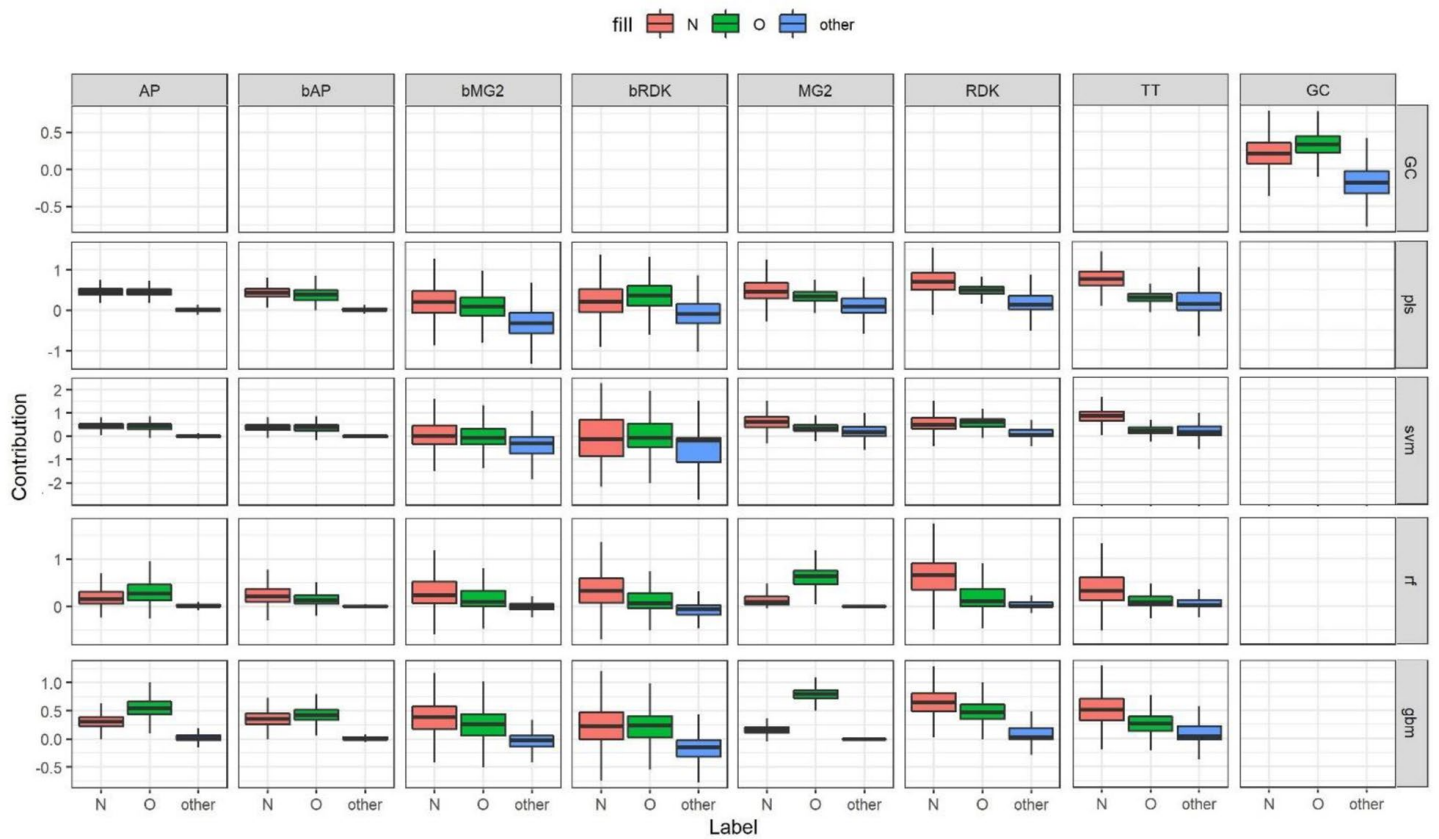
Contributions of nitrogen, oxygen and other atoms for models trained on the N + O data set

This data set is a special case constructed to investigate how interpretation approach and underlying model assign contributions to correlated patterns. In this case models can assign equal or similar contributions to both correlated patterns or prioritize one over the other. PLS and SVM models mainly resulted in comparable contributions for nitrogen and oxygen (Fig. 9). RF and GBM models were more inclined to prioritize one of them. Models trained on binary fingerprints resulted in more balanced contributions than models trained on count-based descriptors. The most striking example was GBM and RF models trained on MG2, where oxygen atoms received much higher contributions than nitrogens.

**Fig. 10:**
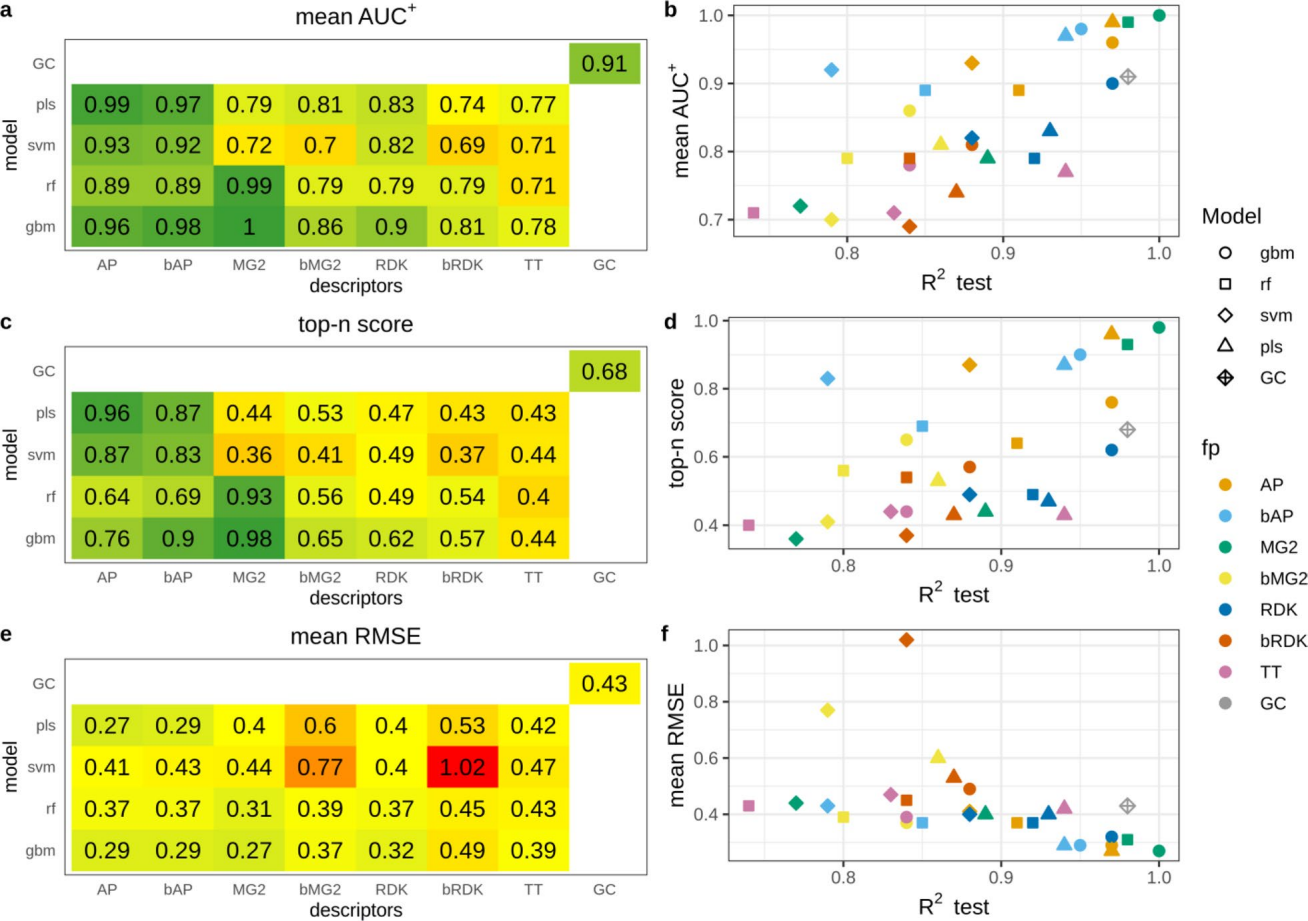
Interpretation performance of models trained on N + O data set. Both N and O atoms were considered as positive patterns

**Fig. 11:**
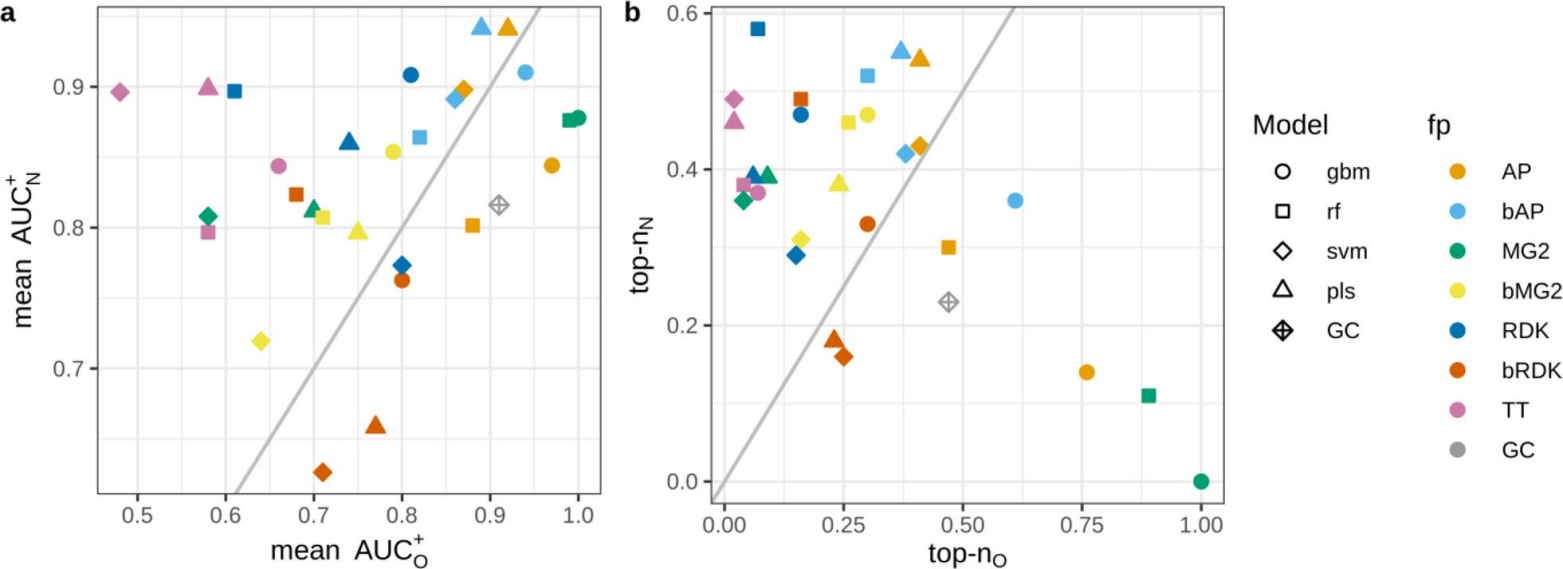
Interpretation performance for the N + O data set, where N or O atoms mutually exlusively were considered true patterns

If we consider both N and O atoms as true patterns the overall interpretation performance is high for models trained on AP and bAP, GBM and RF models trained on MG2, and GC model (Fig. 10). However, not all of these models had high predictive performance. For example, SVM models trained on AP, bAP and MG2 had relative low R^2^_test_—0.75–0.81. We also calculated AUC and top-n scores separately for cases where only O or N atoms were (mutually exclusively) considered true patterns. The closer the points in Fig. 11 to the diagonal, the more balanced assigned contributions. It should be noted that overall interpretation performance does not correlate with interpretation performance calculated for cases where only one of two correlated patterns was considered true. For example, GBM/MG2 model showed perfect performance to retrieve O as true patterns (AUC_O_^+^ = 1.0, top-n_O_ = 1.0) but low performance retrieving N (AUC_N_^+^ = 0.87, top-n_N_ = 0.0). At the same time overall interpretation performance when both patterns were considered true was very high (AUC^+^ = 1.0, top-n = 0.98). This can be easily explained if one will look at assigned contributions. Oxygen atoms received consistently higher contributions among all atoms (Fig. 9). Thus, these atoms were always on top and statistics was perfect. Nitrogen atoms received contributions much lower than oxygens but they were still almost always greater than contributions of remaining atoms. Nitrogens were close to top but not on top and thus AUC value was at a reasonably high level but top-n score was zero because all top scored atoms were oxygen. Considering both patterns as positive resulted in high performance because both were well separated from the rest.

## Discussion

Interpretation performance was reasonable on data sets studied. For highly accurate models SPCI approach retrieved expected patterns with high accuracy. The relationship between prediction and interpretation performance was established in all cases except classification amide data set, where all models had nearly perfect predictive ability, but many of them had relatively poor interpretation results. This can be explained by the presence of multiple true patterns in active molecules, making major impact on interpretation performance. This is a common issue for all similar interpretation approaches based on masking of atoms (similarity maps or matched molecular pairs). Interpretation approaches based on calculating contributions of individual descriptors should be less affected by this issue, but they require interpretable descriptors.

The classification amide data set most notably demonstrated the weakness of AUC as the measure of interpretation accuracy. AUC indicates the ability of models to rank true pattern high. But there is a little difference in AUC values between models which score true patterns on top and those which score true patterns high but not exactly on top. In the latter case it may be difficult to identify true patterns among other highly scored ones. For example, AUC^+^ for GBM and RF models trained on MG2 were 0.98 and 0.94 respectively, but corresponding top-n scores were 0.81 and 0.60 indicating that probability to find true patterns in the top was much higher for the GBM model. Therefore, the proposed top-n score looks a more practically relevant measure of interpretation accuracy.

Among regression models GBM trained on count-based atom pairs or Morgan descriptors had consistently high interpretation performance on all data sets. RF models trained on the same descriptors conceded them a little. The same was observed for classification amide task but not for the pharmacophore task, where the fingerprints type was the most important parameter regardless of modelling algorithm (AP and bAP produced the best results).

It was not surprising that in regression cases binary descriptors often resulted in models with poorer predictive ability than count-based ones. The same was observed for interpretation performance. For classification models there was less discrepancy in both predictive and interpretation performance between count-based and binary descriptors.

While interpretation of conventional models resulted in mostly explainable performance, interpretation performance of GC models raised a question. GC was among models with the highest predictive ability and one may expect highly accurate interpretation results. However, in many cases there was a large difference between interpretation performance (mainly in top-n scores) for GC and conventional models of comparable predictive ability. This was most apparent for regression tasks. We can suggest two possible explanations:

- The implemented interpretation approach is not fully suitable for GC models. It is difficult to prove whether a particular interpretation approach is suitable or not for a particular model, but comparison of interpretation performance with other models may shed some light. For classification tasks in contrast to regression we did not observe large discrepancy in top-n scores between GC and conventional models of similar predictive performance. This indirectly confirms validity of the chosen interpretation approach and its applicability to GC models. In future it would be reasonable to compare results obtained in this study with interpretation results of “orthogonal” interpretation approaches.
- Hidden bias in data sets. It is impossible to control all possible biases in data sets. Conventional models which had high predictive ability comparable to the corresponding GC models demonstrated much better ability to identify true patterns. An explanation may lie in the nature of GC models which learn sophisticated internal representation of molecular structure to find correlation with a given end-point. Thus, these models may construct overly complex patterns correlated with true ones and the end-point. As we demonstrated with the example of N + O data set, GC models assign comparable contributions to correlated features. This can make it difficult to distinguish true patterns from correlated ones. In other words, GC models may “create” a bias and capture it. This ability of GC models can bring some advantages if the underlying true patterns are really highly complex. For tasks of biological activity prediction GC models did not demonstrate systematically better predictivity than conventional models [30]. Thus, constructing complex internal representations may not be necessary to capture relevant structure–activity relationships.

Interpretation of data sets where correlated patterns can appear should be performed with care. In general, it is the desirable ability of models and interpretation approaches to assign comparable contributions to correlated features. If correlated features are true ones this will simplify analysis and interpretation of a model. However, if a model prioritizes only one of correlated features this can result in incomplete interpretation. The same can happen if one removes highly correlated features before model building and does not take them into account at the interpretation stage. At least it may be reasonable to assign the same contributions to correlated features to partially consider them.

Atom-based interpretation, despite its simplicity and attractiveness, does not always result in good performance as it was shown in the case of the pharmacophore data set. This can be explained by the fact that removing one atom may be a too small change in a molecule to be well captured by models. As we observed for the N − O data set, neighbors of true atoms can be confused with them. In this case it would be beneficial to use fragment-based interpretation which can provide a way to capture an approximate position of the true pattern that may be enough for practical applications. For example, if one knows that a particular group causes metabolic instability of a compound, the compound can be modified accordingly even without knowing the exact site of metabolism.

## Conclusions

We created six synthetic data sets of three complexity levels for benchmarking QSAR model interpretation methods. Using these data sets, we investigated an extensive set of descriptor/algorithm combinations and the universal interpretation approach SPCI. We established that interpretation performance may decrease faster than predictivity, and in some cases models with acceptable predictive ability may result in poor interpretation performance. In particular, this was observed for the pharmacophore data set, where models with balanced accuracy on the test set greater than 0.7 had AUC^+^ values close to 0.5. This leads us to conclusion similar to Sheridan [16] that only highly predictive models may reach high interpretation accuracy. However, high predictive ability of models does not guarantee high interpretation accuracy. Graph convolutional models had high predictivity, but on regression tasks they resulted in worse interpretation performance than other models of comparable predictive ability. We hypothesize that GC models learned too complex internal representation resulting in some interpretation bias due to possible appearance of correlated features.

We specifically investigated the issue of capturing of correlated features with the N + O data set. It was established that GC models assign comparable contributions to correlated features. In general, this is the desired ability of models and interpretation approaches. If correlated features are true ones this simplifies analysis; but, if true patterns are randomly correlated with other ones this makes it difficult to distinguish them. Thus, learning of overly complex representation may decrease interpretation performance.

Investigation of three metrics proposed for evaluation of interpretation performance demonstrated that AUC metric is not very sensitive to small changes in ranks of top scored patterns and overly optimistic. Alternative top-n (bottom-n) score is a more stringent and practically reasonable criterion.

We demonstrated a downside of the interpretation approach which virtually removes a particular substructure to estimate its contribution. For classification tasks the presence of multiple identical true patterns in the same molecule leads to poor discrimination between true and false patterns and lower AUC and top-n scores. Any approach based on masking (occlusion) of molecular substructures will be affected by this issue.

We expect that benchmark data sets developed and metrics proposed will be useful for validation and comparison of existing and emerging interpretation methods. The question of transferability of results to real-world applications is always open. We assume that high performance achieved on these benchmarks supports positive conclusion about the method’s validity, and low performance allows to screen out invalid methods. The latter can be inferred with higher confidence: if the method doesn’t work on simple synthetic datasets it is not expected to work on more complex ones. We also anticipate that this work will stimulate investigation of decision making of models, in particular neural networks, since synthetic data sets provide a more controlled environment for such studies.

## Supplementary Information

**Additional file 1: Table S1.** Correlations between count of patterns of interest for molecules of each regression data set and counts of the most common chemical elements. **Figure S1.** Distributions of endpoints in datasets. **Figure S2.** Class-wise distributions of hydrogen bond donors and acceptors for the pharmacophore dataset. **Figure S3.** All 6 datasets (dark-blue points designate molecules) embedded in binned t-SNE plot (1) generated from ChEMBL23 database using the GPU-based t-SNE implementation (2). Original feature space for t-SNE: 2048-dimensional MHFP6 fingerprints (3); perplexity: 50. Number of bins: 50*50. **Figure S4.** Architecture of Graph convolutional network. **Figure S5.** The procedure of removing atoms when interpreting Graph convolutional network. **Figure S6.** Distribution of predicted class probabilities by GC model for the pharmacophore data set.

## Data Availability

Project name: iBenchmark. GitHub: https://github.com/ci-lab-cz/ibenchmark. Operating system(s): cross-platform. Programming language: Python 3. Other requirements: RDKit 2017.09 or higher. License: MIT. Any restrictions to use by non-academics: no. Calculated atomic contributions for all data sets and models—https://doi.org/10.6084/m9.figshare.14484567.v1.
