# Supplementary material for: Benchmarks for interpretation of QSAR models
Source: J Cheminform. 2021 May 26;13:41. doi: 10.1186/s13321-021-00519-x (PMC8157407; doi:10.1186/s13321-021-00519-x)
Supplement: Supplementary file 1 — Additional file 1: Table S1. Correlations between count of patterns of interest for molecules of each regression data set and counts of the most common chemical elements. Figure S1. Distributions of endpoints in datasets. Figure S2. Class-wise distributions of hydrogen bond donors and acceptors for the pharmacophore dataset. Figure S3. All 6 datasets (dark-blue points designate molecules) embedded in binned t-SNE plot (1) generated from ChEMBL23 database using the GPU-based t-SNE implementation (2). Original feature space for t-SNE: 2048-dimensional MHFP6 fingerprints (3); perplexity: 50. Number of bins: 50*50. Figure S4. Architecture of Graph convolutional network. Figure S5. The procedure of removing atoms when interpreting Graph convolutional network. Figure S6. Distribution of predicted class probabilities by GC model for the pharmacophore data set. [file 13321_2021_519_MOESM1_ESM.pdf]

Supporting information:

Benchmarks for interpretation of QSAR models

Mariia Matveieva, Pavel Polishchuk*

Institute of Molecular and Translational Medicine, Faculty of Medicine and Dentistry, Palacky University and University Hospital in Olomouc, Hnevotinska 5, 77900 Olomouc, Czech Republic

pavlo.polishchuk@upol.cz

Table S1. Correlations between count of patterns of interest for molecules of each regression data set and counts of the most common chemical elements.

Dataset	Pattern 1 (SMARTS)	Pattern 2 (SMARTS)					
		[C,c]	[O,o]	[S,s]	[N,n]	Cl	Br
N	[N,n]	0.09	-0.13	0.07	1	-0.02	-0.04
N-O	[N,n]	0.09	0.02	0.02	1	-0.04	-0.07
	[O,o]	0.06	1	0.07	0.02	-0.1	-0.04
N+O	[N,n]	0.23	1	0.1	1	-0.11	0.0
Amide	NC=O	0.09	0.3	0.11	0.25	0.04	0.02

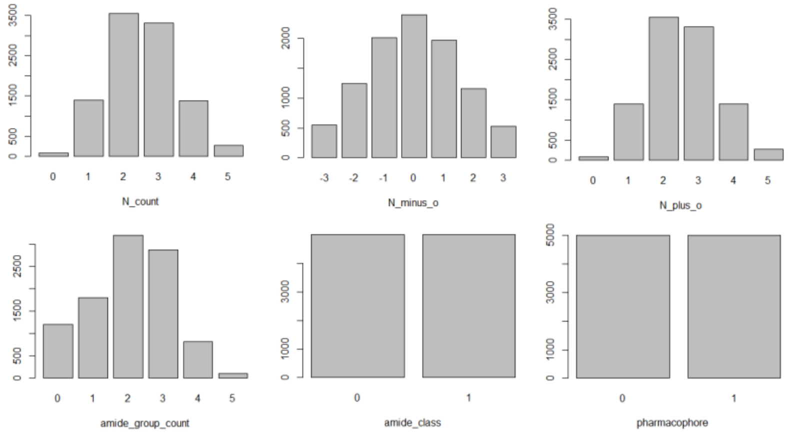

Figure S1. Distributions of endpoints in datasets

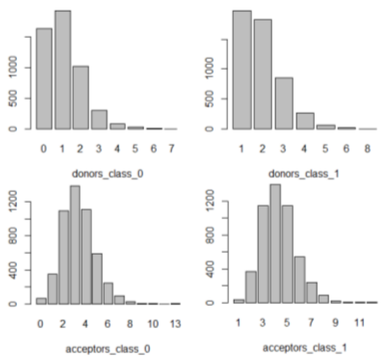

Figure S2. Class-wise distributions of hydrogen bond donors and acceptors for the pharmacophore dataset

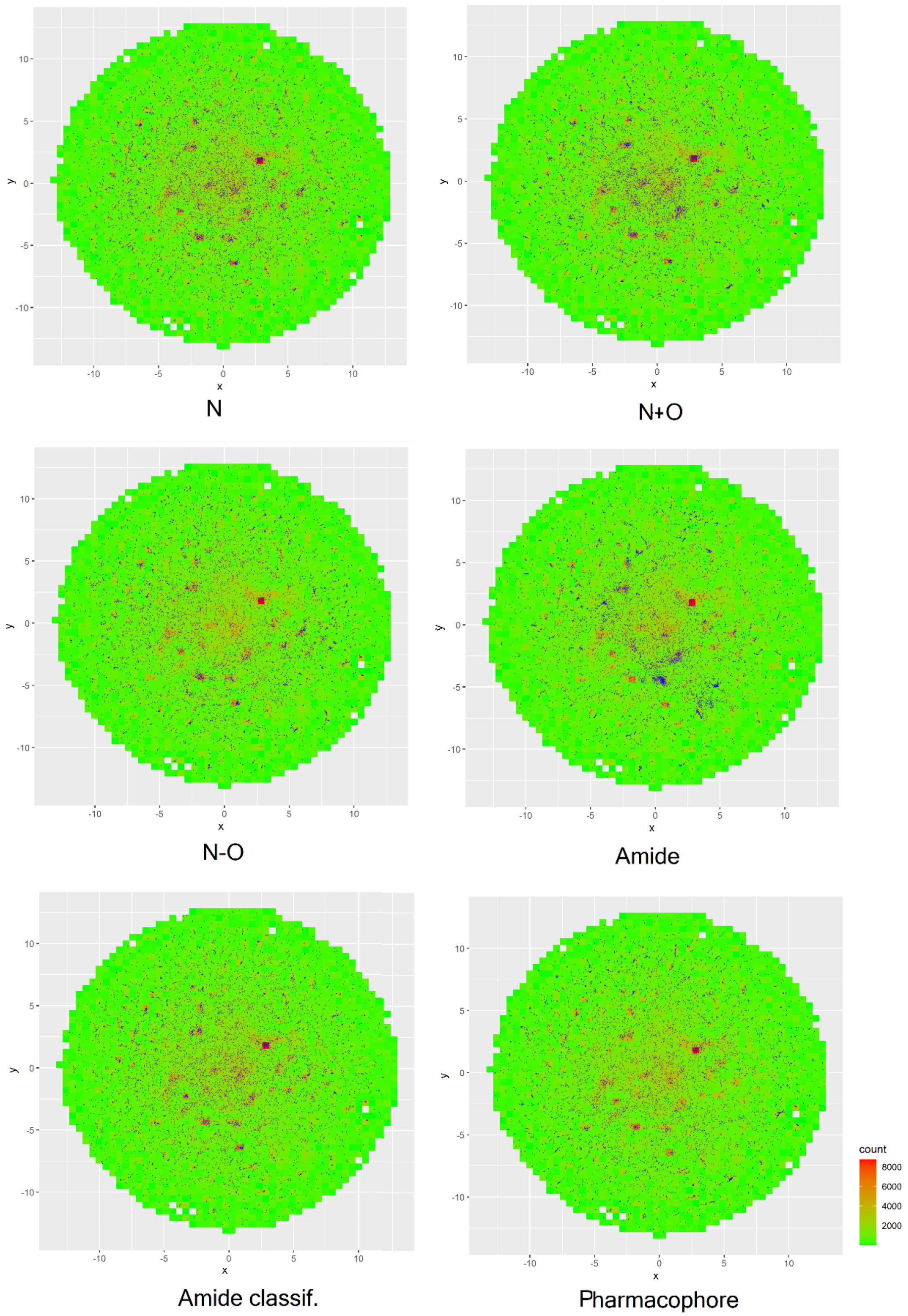

Figure S3. All 6 datasets (dark-blue points designate molecules) embedded in binned *t-SNE* plot (1) generated from ChEMBL23 database using the GPU-based t-SNE implementation (2). Original feature space for *t-SNE*: 2048-dimensional MHFP6 fingerprints (3); perplexity: 50. Number of bins: 50*50.

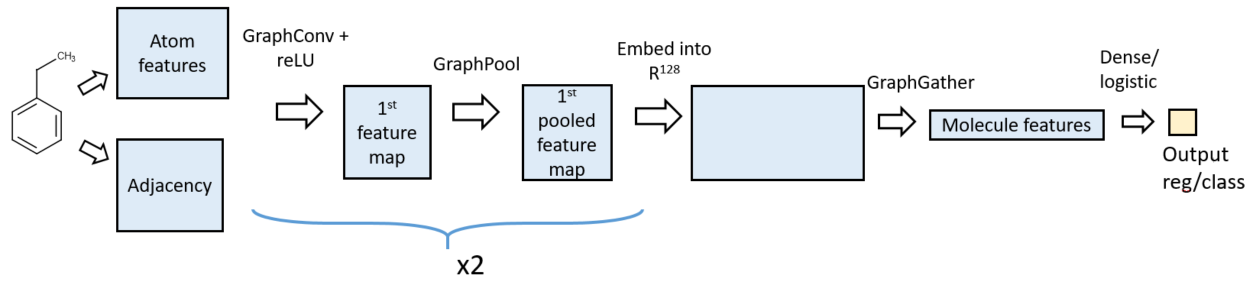

Figure S4. Architecture of Graph convolutional network

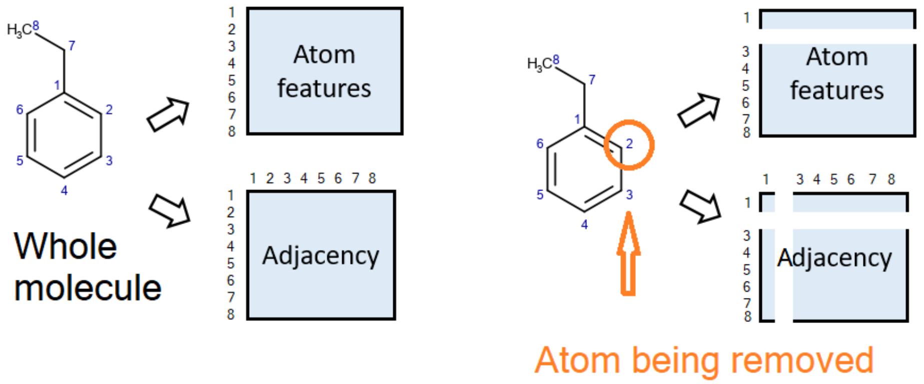

Figure S5. The procedure of removing atoms when interpreting Graph convolutional network

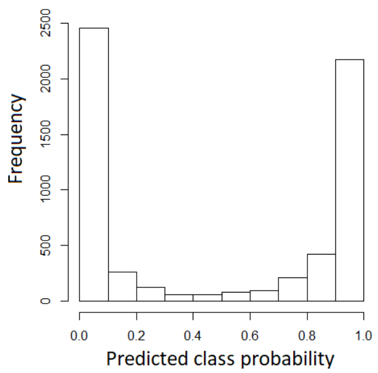

Figure S6. Distribution of predicted class probabilities by GC model for the pharmacophore data set

1. van der Maaten L, Hinton G. (2008) Visualizing Data using t-SNE. *Journal of Machine Learning Research* **9:** 2579-2605.
2. Chan D, Rao R, Huang F, Canny J. (2018) t-SNE-CUDA: GPU-Accelerated t-SNE and its Applications to Modern Data. *2018 30th International Symposium on Computer Architecture and High Performance Computing (Sbac-Pad 2018)***:** 330-338.
3. Probst D, Reymond J. (2018) A probabilistic molecular fingerprint for big data settings. *Journal of Cheminformatics* **10**.
